# Lack of association between *IL-10* and *IL-18* gene promoter polymorphisms and Parkinson’s disease with cognitive impairment in a Chinese population

**DOI:** 10.1038/srep19021

**Published:** 2016-02-02

**Authors:** Zhenhua Liu, Jifeng Guo, Yaqin Wang, Kai Li, Jifeng Kang, Yang Wei, Qiying Sun, Qian Xu, Changshui Xu, Xinxiang Yan, Beisha Tang

**Affiliations:** 1 Department of Neurology, Xiangya Hospital, Central South University, Changsha, 410008 Hunan, People’s Republic of China; 2 State Key Laboratory of Medical Genetics, Changsha, 410008 Hunan, People’s Republic of China; 3 Key Laboratory of Hunan Province in Neurodegenerative Disorders, Central South University, Changsha, 410008 Hunan, People’s Republic of China; 4 Neurodegenerative Disorders Research Center, Central South University, Changsha, 410008 Hunan, People’s Republic of China; 5 Department of Neurology, Henan provincial people’s hospital, Zhengzhou, 450003, Henan, People’s Republic of China

## Abstract

Inflammatory processes have been implicated in the pathogenesis of Parkinson’s disease (PD), including the development of PD-associated cognitive impairment. Whether genetic variants of inflammatory cytokine genes influence the risk of cognitive impairment in PD is unknown. In this study, we investigated single nucleotide polymorphisms (SNPs) in the *IL-10* promoter (rs1800871 and rs1800872) and in the *IL-18* promoter (rs1946518 and rs187238) in a Han Chinese cohort (N = 933). PD patients (N = 460) and controls (N = 473) were genotyped. Additionally, 268 PD patients were divided into three subgroups [cognitively normal (PD-NC), mild cognitive impairment (PD-MCI), and with dementia (PD-D)] on the basis of their performance on a battery of neuropsychological tests. No associations were found between the aforementioned polymorphisms and cognitive impairment in PD; thus no confirmatory evidence for the hypothesis of *IL-10* and *IL-18* alleles modulating the risk of cognitive impairment in Chinese PD patients was obtained.

Parkinson’s disease (PD) is the second most common neurodegenerative disorder affecting geriatric patients, after Alzheimer disease (AD). Its clinical manifestations include a resting tremor, rigidity, bradykinesia, and postural instability. Additionally, PD patients often suffer cognition dysfunction that can affect their quality of life severely. Increasing evidence suggests that mild cognitive impairment in PD (PD-MCI) represents the initial stage of a progressive cognitive decline leading to PD dementia (PD-D), which occurs eventually in as many as 80% of PD patients^1^,^2^. At present, the pathophysiology of PD-associated cognitive decline is poorly understood and an optimal clinical management strategy for the condition has not been delineated.

Etiologically, PD is complex and thought to be a heterogeneous disease caused by both environmental and genetic factors. Inflammatory processes have been implicated in the pathogenesis of PD^3^,^4^. Interest in interactions between inflammatory cytokines and PD has spurred genetic studies examining potential associations between cytokine gene polymorphisms and PD. Cytokine genes of interest have included the interleukin (IL)-10 (*IL-10*), IL-18 (*IL-18*), tumor necrosis factor-alpha (*TNFα*), and IL-1-beta (*IL-1β*) genes, among others^5^,^6^,^7^.

It is noteworthy that cytokines have also been shown to be involved in other neurodegenerative disorders, including Alzheimer disease (AD)^8^. Impairments in inflammatory processes have been shown to result in cognition dysfunction and numerous studies have linked particular inflammatory cytokines with cognitive deterioration in AD^9^,^10^,^11^. Similarly, Reale and colleagues found that peripheral blood monocytes isolated from PD patients release abnormally high levels of several cytokines, including interleukin (IL)-10, suggesting that elevated serum levels of cytokines may be due directly to immunological dysregulation, rather than being secondary to degeneration of dopaminergic neurons^12^.

IL-10 is a key anti-inflammatory cytokine, encoded by the *IL-10* gene located at 1q31–32. The *IL-10* promoter is highly polymorphic and IL-10 expression is known to be affected robustly by two single nucleotide polymorphisms (SNPs), located 819 and 592 nucleotides before the transcription initiation site^13^. Genetic association studies have indicated that the −819 T/C (rs1800872) and −592 A/C (rs1800871) SNPs in the *IL-10* promoter are linked to multiple diseases, including AD and schizophrenia, as well as PD^14^,^15^.

IL-18, a member of the IL-1 superfamily, is a pro-inflammatory cytokine that functions in the inflammatory cascade. The *IL-18* gene is located at 11q22.2–22.3 and its promoter region contains multiple transcription initiation start sites. Two SNPs, namely −607C/A (rs1946518) and −137G/C (rs187238), have been shown to correlate with IL-18 production^3^. *IL-18* polymorphisms have been reported to be associated with both PD and AD^6^,^11^.

In light of the aforementioned findings, we hypothesized that the inflammatory cytokines IL-10 and IL-18 may also play important roles in PD-associated cognitive impairment. Here, we explored whether the −819T/C and −592A/C SNPs of *IL-10* and the −607C/A and −137G/C SNPs of *IL-18* affect cognitive performance in Han Chinese patients with PD.

## Results

### Demographics, clinical, and neuropsychological assessments

The demographics and clinical characteristics of the PD and healthy control groups are summarized in Table 1. The demographics and clinical characteristics of cognitively normal PD patients (PD-NC), PD patients with mild cognitive impairment (PD-MCI), and PD patients with dementia (PD-D), classified into these subgroups based on comprehensive neuropsychological testing results, are shown in Table 2.

### Cytokine gene polymorphisms and PD risk

One of the four SNPs selected for screening, −607 C/A (rs1946518) in *IL-18*, deviated significantly (*p* < 0.05) from Hardy-Weinberg equilibrium (HWE) in both the PD group and the control group. The genotype and allele distributions of the remaining three studied SNPs (*IL-10* rs1800871, *IL-10* rs1800872, and *IL-18* rs187238) in PD patients and healthy controls are reported and compared in Table 3. After adjustment for sex and age, logistic regression analyses revealed no significant differences in genotype or allele distribution between the PD group and the HC group for *IL-10* rs1800871, IL-10 rs1800872, or *IL-18* rs187238 (Table 3).

### Association of cytokine gene polymorphisms with cognitive status and impairment severity in PD patients—PD subgroup comparisons

After adjustment for age, gender, education, disease duration, and disease severity, logistic regression analyses revealed no significant differences in genotype or allele distribution for the studied SNPs between PD patients with cognitive impairment (PD-D and PD-MCI subgroups combined) and the PD-NC subgroup (Table 4). Likewise, similarly adjusted regression analyses showed that genotype and allele frequencies were similar between the PD-D and PD-MCI subgroups (Table 4).

## Discussion

In the present study, we report the results of the first formal examination, to our knowledge, of whether *IL-10* and *IL-18* promoter SNPs are associated with cognitive impairment in PD. We conducted logistic regression analyses of two functional variants [−819 T/C (rs1800872) and −592 A/C (rs1800871)] in the promoter region of *IL-10* and one functional variant [−137 G/C (rs187238)] in the promoter region of *IL-18* to assess their relationships with sporadic PD and PD-associated cognitive decline in a Han Chinese population. We found no significant differences in genotype or allele distributions between the PD group and the HC group for the *IL-10* −592 A/C, *IL-10* −819 T/C, and *IL-18* −137 G/C SNPs. Furthermore, we did not obtain evidence in support of an association between these SNPs and cognitive status (as evidenced by comprehensive neuropsychological testing) in PD patients. Rather, the genotype and allele distributions in PD patients with cognitive impairment (severe PD-D and PD-MCI subgroups) were similar to the distributions observed for the cognitively normal PD patients (PD-NC subgroup), and the genotype and allele distributions in the more severely impaired PD-D subgroup were similar to those in the relatively more mildly impaired PD-MCI group. Hence, the results of our study indicate that these three SNPs do not modulate susceptibility to PD or PD-associated cognitive impairment in ethnic Han Chinese people.

Recent studies have highlighted a crucial role for cytokines in PD^16^. Genetic polymorphisms in cytokine genes, especially in regulatory regions, are in a position to influence the expression of these cytokines, and thus regulate the intensity of immune responses. Thus, it is reasonable to suppose that such polymorphisms may affect the pathogenesis of neurodegenerative disorders^17^. Although this supposition has been confirmed for AD^8^, a potential similar association between inflammation-related gene polymorphisms and cognitive impairment has been studied less in PD.

The anti-inflammatory cytokine IL-10, which is produced primarily by monocytes and lymphocytes, carries out multiple immunoregulatory functions, including moderating the biosynthesis of pro-inflammatory cytokines and suppressing cellular immunity in the central nervous system, favoring neural and glial cell survival. Moreover, given that the −819 T/C (rs1800872) and −592 A/C (rs1800871) SNPs in *IL-10* have been shown to affect IL-10 levels together with prior data indicating that IL-10 production is elevated in PD patients^12^,^13^,^14^,^15^, our present negative findings regarding these SNPs and cognitive status in PD are somewhat surprising.

Meanwhile, prior evidence has suggested that IL-18 may have direct influences on neuronal viability and neurodegeneration. For example, plasma and cerebrospinal fluid levels of IL-18 have been reported to be elevated in AD patients, and stimulated peripheral blood mononuclear cells from AD patients produce more IL-18 than analogous cells from control subjects^18^. IL-18 levels have been reported to be significantly higher in PD patients than in HC subjects, and the two *IL-18* SNPs selected for study here have been shown to influence *IL-18* activity^12^,^19^.

Our study has a number of possible limitations. Firstly, because the research was performed with a mainland Chinese cohort, we do not know whether the results will extend to populations of other ethnic origins. Secondly, no significant associations between cytokine polymorphisms and PD with cognitive impairment have been found in the Chinese population. Thirdly, the sample sizes for our PD subgroups (PD-NC, PD-MCI, and PD-D) were relatively small for an association study. Thus, although we did not observe suggestive trends, it is possible that a study with larger subgroups, and thus greater statistical power, might reveal a significant association between genotype or allele frequency of the presently examined SNPs and cognitive performance among PD patients. A larger study should be performed to confirm the present negative results. Another limitation of this study was the lack of data regarding plasma levels of IL-10 and IL-18; however the functional relevance of the studied polymorphisms is well known as described above.

In conclusion, our results indicated that *IL-10* and *IL-18* promoter-region SNPs are not associated with cognitive impairment in Han Chinese PD patients. Further association studies with larger sample sizes and encompassing heterogeneous populations are needed to confirm whether or not cytokine gene polymorphisms play a role in the pathogenesis of PD cognitive impairment.

## Methods

### Ethics statement

The study was conducted in accordance with the Declaration of Helsinki and was approved by the ethics committee of Central South University. All participants provided informed consent before participating in the study.

### Study population

460 mainland Chinese sporadic PD patients were collected from the outpatient neurology clinics of Xiangya Hospital during 2009–2014. The diagnosis of PD was made by 2 or more experienced neurologists according to the United Kingdom brain bank criteria^20^. A control group of 473 healthy mainland Chinese subjects from the same geographic areas was obtained, matched for age and sex.

### Clinical assessment

Demographic and clinical assessment of motor function, including Unified Parkinson’s Disease Rating Scale (UPDRS) motor scores and Hoehn and Yahr (H-Y) stage, was obtained by trained examiners.

### Assessment of cognition

In this study, only 268 PD patients and 282 Healthy controls underwent a series of neuropsychological assessments. Neuropsychological assessments were administered by trained research staff. Protocol of neuropsychological testing included: Mini-Mental State Examination(MMSE), the Montreal Cognitive Assessment (MoCA), Digit span test (DST), Stroop Color-Word test (SCWT), Frontal Assessment Battery (FAB), Judgment of Line Orientation test (JOLT), Clock-drawing test (CDT), Hopkins Verbal Learning Test (HVLT), Boston Naming Test, the Wechsler Memory Scale (WMS), Hamilton Depression Scale (HAM-D), Apathy scale (AS), Neuropsychiatric Inventory (NPI). All tests were recommended by the Movement Disorders (MDS) Task Force^21^.

### Diagnostic criteria for PDD and PD-MCI

Dementia diagnosis was based on the MDS Task Force criteria^21^. PD-MCI cases had unimpaired functional activities of daily living but scored 1.5 SDs or more below normative data on at least two measures within at least one of the five MDS Task Force cognitive domains (executive function; memory; attention, working memory and speed of processing; and visuospatial)^22^.

### DNA extraction and genotyping

Blood samples were obtained from all the patients, and genomic DNA was extracted from peripheral blood lymphocytes using standard phenol-chloroform procedures. DNA was extracted from full blood or buffy coat using standard techniques. SNP was genotyped by matrix-assisted laser desorption/ionization–time-of-flight mass spectrometry (MALDI-TOF MS) using the MassArray system (Sequenom) as described^23^. For quality control, the positive and negative controls (with no DNA) were included on every 96-well assay plate, and 50 patients and 50 controls were randomly selected for Sanger sequencing. Briefly, locus-specific polymerase chain reaction and detection primers were designed using the MassArray Assay Design 3.0 software (Sequenom). Approximately 15 ng of genomic DNA was used to genotype each sample. The sample DNAs were amplified by primers flanking the targeted sequence, followed by dephosphorylation and allele-specific primer extension. Cleaned extension products were loaded into a 384-format Spectro-Chip and subjected to MALDI-TOF mass spectrometry. Finally, the resultant data were processed and analyzed by the Sequenom MassArray Typer software (Sequenom).

### Statistical analysis

Statistical analysis was performed with SPSS 19.0 software (SPSS Inc., Chicago, IL, USA). Hardy-Weinberg equilibrium (HWE) was examined. We used logistic regression analysis to test for the association between the studied polymorphisms and PD risk or PD cognitive impairment. The estimated odds ratios (ORs) and relative 95% confidence intervals (CIs) were adjusted for age, gender, education, disease duration and disease severity(UPDRS-III and Hoehn–Yahr stage). The Odds Ratios (OR) and 95% confidence intervals (95% CI) were calculated. A two-tailed test p-value of <0.05 was considered statistically significant.

## Additional Information

**How to cite this article**: Liu, Z. *et al.* Lack of association between *IL-10* and *IL-18* gene promoter polymorphisms and Parkinson’s disease with cognitive impairment in a Chinese population *Sci. Rep.* **6**, 19021; doi: 10.1038/srep19021 (2016).

## Figures and Tables

**Table 1:** Demographics and clinical data of all patients and health controls. The data in the table were presented in mean and standard deviation (SD).

	PD (n = 460)	HCs (n = 473)
Age (years)	62.15 ± 9.76	58.38 ± 12.03
Sex (male: female)	236:224	237:236
Disease duration (years)	6.58 ± 4.92	—
H-Y	2.45 ± 1.03	—
UPDRS-III	20.29 ± 13.09	—

Abbreviations: PD, Parkinson disease; HCs, Health controls; SD, standard deviation, H-Y, Hoehn and Yahr stage; UPDRS-III, Unified Parkinson’s Disease Rating Scale part III.

**Table 2:** Demographics and clinical data of participants who completed series of neuropsychological assessments in the study. The data in the table were presented in mean and standard deviation (SD).

	PD-NC (n = 92)	PD-MCI (n = 76)	PD-D (n = 100)
Age (years)	65.53 ± 6.45	64.82 ± 7.40	67.96 ± 7.31
Sex (male: female)	50:42	42:32	53:47
Education (years)	11.48 ± 3.30	9.83 ± 3.36	8.78 ± 2.92
Disease duration (years)	5.97 ± 3.13	8.13 ± 3.44	7.01 ± 3.29
H-Y	2.26 ± 1.15	2.36 ± 0.86	2.95 ± 0.92
UPDRS-III	23.89 ± 9.23	27.43 ± 10.49	36.54 ± 12.28
MMSE	28.18 ± 1.64	27.05 ± 1.76	21.00 ± 4.18
MoCA	25.23 ± 1.62	23.00 ± 2.12	17.63 ± 3.61

Abbreviations: PD-D, Parkinson’s disease with dementia; PD-MCI, Parkinson’s disease with mild cognitive impairment; PD-Nc, Parkinson’s disease with normal cognition; H-Y, Hoehn and Yahr stage; UPDRS-III, Unified Parkinson’s Disease Rating Scale part III; MMSE, Mini-Mental State Examination; MoCA, Montreal cognitive assessment.

**Table 3:** Genotype and allele frequencies distribution of *IL-10* and *IL-18* polymorphism in Parkinson’s disease (PD) patients and health controls.

SNP	Genotype/allele	PD(n = 460)	HCs(n = 473)	*p*^a^	OR^a^ (95%CI^a^)
*IL10* (−592 A/C)	C	271(29.5%)	305(32.2%)	0.226	0.884(0.723–1.080)
rs1800871	T	649(70.5%)	641(67.8%)		
	CC	51(11.0%)	47(9.9%)	0.237	0.888(0.730–1.081)
	CT	169(36.7%)	211(44.6%)		
	TT	240(52.3%)	215(45.5%)		
					
*IL10* (−819 T/C)	C	273(28.4%)	305(32.2%)	0.295	0.899(0.736–1.097)
rs1800872	A	647(71.6%)	641(67.8%)		
	CC	53(11.5%)	48(10.2%)	0.309	0.903(0.745–1.098)
	CA	167(36.3%)	209(44.2%)		
	AA	240(52.2%)	216(45.6%)		
*IL18* (−137 G/C)	C	121(13.2%)	113(12.0%)	0.536	1.092(0.826–1.444)
rs187238	G	799(86.8%)	833(88.0%)		
	CC	10(2.2%)	4(0.8%)	0.535	1.093(0.826–1.446)
	CG	101(21.9%)	105(22.2%)		
	GG	349(75.9%)	364(77.0%)		

Abbreviations: PD, Parkinson disease; HCs, health controls; SNP, single-nucleotide polymorphism.

^a^The estimated odds ratios (ORs) and relative 95% confidence intervals (95% CI) were adjusted for gender and age at enrollment.

**Table 4:** Genotype and allele frequencies distribution of *IL-10* and *IL-18* polymorphisms between PD patients with or without cognitive impairment.

SNP	Genotype/allele	PD-NC(n = 92)	PD-MCI(n = 76)	PD-D(n = 100)	PD-D plusPD-MCI(n = 176)	PD-D plus PD-MCI vs PD-NC		PD-D vs PD-MCI		
						*p*^a^	OR^a^(95%CI^a^)	*p* P^a^	OR^a^(95%CI^a^)	
*IL10* (−592 A/C)	C	49 (26.6%)	43(28.3%)	55(27.5%)	98(27.8%)	0.723	0.926 (0.606–1.415)	0.789	1.072 (0.647–1.775)	
rs1800871	T	135 (73.4%)	109(71.7%)	145(72.5%)	254(72.2%)					
	CC	10 (10.4%)	4(5.2%)	12(12.0%)	16(9.1%)	0.736	0.933 (0.622–1.398)	0.796	1.066 (0.656–1.733)	
	CT	29 (31.4%)	35(46.1%)	31 (31.0%)	66(37.5%)					
	TT	53 (58.2%)	37(48.7%)	57 (57.0%)	94(53.4%)					
										
*IL10* (−819 T/C)	C	49 (28.0%)	43(28.3%)	56 (28.0%)	99 (28.1%)	0.896	0.973 (0.644–1.469)	0.698	1.104 (0.669–1.821)	
rs1800872	A	135 (72.0%)	109(71.7%)	144 (72.0%)	253 (71.9%)					
	CC	10(10.9%)	5(6.6%)	13 (13.0%)	18 (10.2%)	0.906	0.976 (0.662–1.438)	1.093 (0.681–1.754)		
	CA	29 (31.5%)	33(43.4%)	30 (30.0%)	63 (35.8%)					
	AA	53 (57.6%)	38(50.0%)	57 (57.0%)	95 (54.0%)					
										
*IL18* (−137 G/C)	C	27 (14.7%)	12 (7.9%)	30(15.0%)	42 (11.9%)	0.572	1.177 (0.669–2.073)	0.053	0.474 (0.222–1.010)	
rs187238	G	157 (85.3%)	140 92.1%)	170 (85.0%)	310 (88.1%)					
	CC	2 (2.2%)	0(0.0%)	4 (4.0%)	4 (2.3%)	0.578	1.171 (0.672–2.041)	0.069	0.511 (0.248–1.055)	
	CG	23 (25.0%)	12 (15.8%)	22 (22.0%)	34 (19.3%)					
	GG	67 (72.8%)	64(84.2%)	74 (74.0%)	138 (78.4%)					

Abbreviations: SNP, single-nucleotide polymorphism; PD-D, Parkinson’s disease with dementia; PD-MCI, Parkinson’s disease with mild cognitive impairment; PD-NC, Parkinson’s disease with normal cognition.

^a^The estimated odds ratios (OR) and relative 95% confidence intervals (95% CI) were adjusted for gender, age, education, disease duration, and disease severity (UPDRS-III and H-Y stage).
